# Coronavirus: the spread of misinformation

**DOI:** 10.1186/s12916-020-01556-3

**Published:** 2020-03-18

**Authors:** Areeb Mian, Shujhat Khan

**Affiliations:** Imperial College London, London, SW7 2AZ UK

**Keywords:** COVID-19, Coronavirus, Misinformation, Internet, Antiscience, Pandemic, Public health

There has been a global rise recently in the spread of misinformation that has plagued the scientific community and public. Disconnect between scientific consensus and members of the public on topics such as vaccine safety, the shape of the earth, or climate change has existed for a number of years. However, this has progressively worsened as society has become further divided in the political climate of today. In turn, it has created an optimal environment for antiscience groups to gain footing and propagate their false theories and information. The public health crisis emerging due to the coronavirus (COVID-19) is also now beginning to feel the effects of misinformation.

We stand with our colleagues Calisher et al., who recently published a statement of solidarity to fight against COVID-19 and to promote scientific evidence and unity over misinformation and conjecture [1]. Just as the coronavirus itself, misinformation has spread far and wide, drowning out credible sources of information. Over the last couple of months, posts from the World Health Organization (WHO) and the US Center of Disease Control (CDC) have cumulatively only achieved several hundred thousand engagements, considerably eclipsed by hoax and conspiracy theory sites, which have amassed over 52 million. This serves to emphasise the popularity of unverified sources of information.

Similarly, misinformation was widespread during the early years of the HIV epidemic. It too was plagued by conspiracy theories, rumours, and misinformation for many years, with the effects still visible in regions to this day. Many people continue to argue that HIV does not exist, or cause AIDS, and that its therapies are toxic to human health. All the arguments proposed by these deniers have been rebuked through a multitude of scientific publications and debate. Yet, they continue to persist. The influence of these false arguments can be so infectious that it can influence governmental policy, which has the potential to be fatal. This was particularly highlighted by the Mbeki South African government’s denialism of HIV in the early 2000s and their infamous rejection of the evidence surrounding the efficacy of HIV medication. In turn, thousands of mothers were denied access to antiretroviral therapies. Instead, the government promoted the unsubstantiated use of herbal remedies including garlic, beetroot, and lemon juice for AIDS treatment [2], leading to unnecessary HIV transmission, especially to children from pregnant mothers. This costs more than 300,000 lives [3]. It is important that we learn from past mistakes, and the media has a large role to play in this. It seems in a bid to increase viewership, major media organisations are creating dramatic headlines but are instead inciting panic amongst the public. Whilst healthcare professionals are still learning about the virus, the media has already begun to speculate about the potential health impact that the virus can have, and by publishing the potential worst effects of the virus, it only serves to fuel panic amongst the general public.

As COVID-19 turns into full-fledged public health crisis, multiple theories regarding the virus’ origin have taken hold on the internet, all with a common theme: the virus was artificially created in a lab by a rogue government with an agenda. This misinformation originated from social media accounts and websites with no credible evidence to support their claims. These posts have amassed over 20 million engagements, rising each day, and the theories continue to gain traction and following on the internet, despite scientists from multiple nations analysing the genome of COVID-19 and coming to the decisive conclusion that the virus originated in nature from an animal source [4, 5]. If powerful and clear statements are not made denouncing and debunking these fabrications, then the impact on the populous has the potential to be devastating.

Furthermore, basic information on how to reduce transmission and exposure to the virus has been muddled by uncredited sources. For example, a popular myth currently circulating is that home remedies can cure or prevent people from getting the virus. Taking vitamin C and eating garlic are being hailed as miracle remedies despite the complete lack of evidence. Whilst many of these are harmless, some have the potential to be very dangerous. One product that has gained traction on social media involves mixing sodium chlorite solution with citric acid, generating chlorine dioxide solution. The instructions then state for this powerful bleaching agent to be consumed, promising antimicrobial, antiviral, and antibacterial actions. The American Food and Drug Administration has previously served severe warnings against this, as it causes severe vomiting, life-threatening low blood pressure, and acute liver failure [6, 7]. Spread of false information drowns out credible sources and in turn results in further public confusion, ultimately leading to greater spread, and inefficient mitigation of virus transmission.

In the face of a pandemic, it is important for governments to be transparent and relay clear, honest information to the public. Public confusion leaves citizens unprepared for combatting a public health crisis. Additionally, it is dangerous for politicians to politicise this pandemic. At times like this, the message from government leaders needs to be consistent so that the public can regain trust in civil servants.

Governments and figures in the media should utilise the knowledge of experts, particularly from the CDC and WHO, to accurately deliver information in a sensible and precise manner so as to not incite panic amongst the public. The appearance of this virus offers an opportunity for the public and medical health professionals to fight in unity against this common threat. If health bodies appropriately manage, educate, and address the people’s concerns, there is an opportunity to bridge the level of distrust that has arisen by antiscience movements in recent times.

## Data Availability

Not applicable
